# Glucose-dependent Insulinotropic Polypeptide (GIP) Resistance and β-cell Dysfunction Contribute to Hyperglycaemia in Acromegaly

**DOI:** 10.1038/s41598-019-41887-7

**Published:** 2019-04-04

**Authors:** Vikram Singh Shekhawat, Shobhit Bhansali, Pinaki Dutta, Kanchan Kumar Mukherjee, Kim Vaiphei, Rakesh Kochhar, Saroj K. Sinha, Naresh Sachdeva, Anura V. Kurpad, Kishor Bhat, Sunder Mudaliar, Anil Bhansali

**Affiliations:** 1 Department of Endocrinology, PGIMER Chandigarh, Sector 12, Chandigarh, 160012 India; 2 Department of Neurosurgery, PGIMER Chandigarh, Sector 12, Chandigarh, 160012 India; 3 Department of Pathology, PGIMER Chandigarh, Sector 12, Chandigarh, 160012 India; 4 Department of Gastroenterology, PGIMER Chandigarh, Sector 12, Chandigarh, 160012 India; 5 Department of Physiology, St. John’s Medical College, Bangalore, India; 6 Department of Medicine, University of California, San Diego, La Jolla, California, USA

## Abstract

Impaired insulin sensitivity (IS) and β-cell dysfunction result in hyperglycaemia in patients of acromegaly. However, alterations in incretins and their impact on glucose-insulin homeostasis in these patients still remain elusive. Twenty patients of active acromegaly (10 each, with and without diabetes) underwent hyperinsulinemic euglycaemic clamp and mixed meal test, before and after surgery, to measure indices of IS, β-cell function, GIP, GLP-1 and glucagon response. Immunohistochemistry (IHC) for GIP and GLP-1 was also done on intestinal biopsies of all acromegalics and healthy controls. Patients of acromegaly, irrespective of presence or absence of hyperglycaemia, had similar degree of insulin resistance, however patients with diabetes exhibited hyperglucagonemia, and compromised β-cell function despite significantly higher GIP levels. After surgery, indices of IS improved, GIP and glucagon levels decreased significantly in both the groups, while there was no significant change in indices of β-cell function in those with hyperglycaemia. IHC positivity for GIP, but not GLP-1, staining cells in duodenum and colon was significantly lower in acromegalics with diabetes as compared to healthy controls possibly because of high K-cell turnover. Chronic GH excess induces an equipoise insulin resistance in patients of acromegaly irrespective of their glycaemic status. Dysglycaemia in these patients is an outcome of β-cell dysfunction consequent to GIP resistance and hyperglucagonemia.

## Introduction

Acromegaly is characterized by chronic growth hormone (GH) excess and almost invariably is caused by a GH-secreting pituitary adenoma^1^. Metabolic complications like disorders of glucose metabolism are frequently associated with acromegaly^2–4^. The prevalence of glucose intolerance in acromegaly has been reported to range between 19–56% in various studies, and upto 20% may have diabetes at diagnosis^3–6^. The metabolic actions of GH are mainly diabetogenic, and it is a potent antagonist of the insulin action on carbohydrate metabolism^5,6^. The key pathogenetic mechanism for GH-induced glucose intolerance is insulin resistance (IR)^7,8^ and it is mediated by GH-induced increased lipolysis, inhibition of the post-receptor insulin signalling by blockade of insulin receptor substrate-1 (IRS-1) and phosphatidylinositol 3-kinase (PI3K) signalling, induction of suppressor of cytokine signalling pathway, and decreased expression of adiponectin and visfatin^4,9–19^. Impaired β-cell function and insulin secretion have also been implicated to contribute to glucose intolerance in these patients^20,21^. Studies done in the past have shown that insulin sensitivity (IS) is reduced to comparable levels in patients of acromegaly with and without glucose intolerance, suggesting that a GH-mediated compensatory hyperfunction of pancreatic β-cells might counterbalance the reduced IS in patients with normal glucose tolerance, but not in those with diabetes^22^.

The incretin hormones strongly influence the glucose-insulin homeostasis, however they have not been explored extensively and little is known about their effect on carbohydrate metabolism in patients with acromegaly. While there are only a couple of studies investigating glucose-dependent insulinotropic polypeptide (GIP) and glucagon levels in acromegaly, there are no studies documenting the alterations in glucagon like peptide-1 (GLP-1) levels in patients with acromegaly^23,24^. Further, the medical therapies such as somatostatin analogues used in the treatment of acromegaly can also influence the glucose-insulin homeostasis^25,26^. Hence, exploring the incretin-axis in patients with acromegaly will not only help us to understand the pathogenesis of glucose intolerance in these patients, it may also help to manage them more effectively. Our study aimed to investigate the alterations in incretin axis in patients with active acromegaly both before and after surgery. We also explored the correlation between the circulating levels of incretins with expression pattern of GIP and GLP-1 staining cells in the intestinal biopsies of these patients.

## Methods

### Subjects and study design

Our study was a prospective case-control study. It included 20 treatment-naïve patients of active acromegaly (10 with diabetes and 10 without diabetes) with elevated age–matched IGF-1, GH nadir during OGTT >1.0 ng/ml and MRI evidence of pituitary adenoma. Patients were excluded if they harbored any chronic illness including cardiovascular, chronic hepatic or renal disease. Once the eligibility criteria were confirmed, a written informed consent was obtained from all patients. The study was approved by the Institute Ethics Committee. Complete blood count, biochemistry, fasting and post-prandial plasma glucose, HbA1c, anterior pituitary hormones, IGF-1, fasting plasma insulin (FPI), C-peptide and MRI-sella were done in all study subjects. Patients with deficiency of any anterior pituitary hormone/s were adequately replaced. Those with hyperglycaemia were treated with metformin and insulin therapy. All patients in the study group, both with euglycaemia and those with hyperglycaemia (after achieving HbA1c <8.0%) underwent a hyperinsulinemic euglycaemic clamp to assess IS, and a three hour mixed meal test (MMT) to measure insulin, C-peptide for calculating various indices of IS and β-cell function. These tests were done in random order after an overnight fast of 12 hours within a 1-to 2-week interval with minimum interval of 7 days between the two tests. All long-acting and short- acting insulin, and metformin were stopped at least 3, 1 and 7 days, respectively, before these tests. To investigate the incretin response, we also measured GIP (total), GLP-1 (total) and glucagon during the MMT. Further, 10 healthy controls also underwent MMT to measure GIP (total), GLP-1 (total) and glucagon for comparison with the study population. Before surgery all patients underwent upper gastro-intestinal endoscopy and colonoscopy during which duodenal and sigmoid biopsies were taken to examine for the expression pattern of cells staining for GIP and GLP-1, using immunohistochemistry (IHC). Twenty age, BMI and HbA1c matched controls were also enrolled to compare the findings of intestinal biopsies with acromegalic subjects.

All patients underwent transsphenoidal surgery (TSS) to extirpate the pituitary adenoma. Both HEC and MMT were again repeated 3 months following TSS in all patients to document the effect of surgery on the indices of IS, β-cell function and incretin hormone response.

### Biochemical analysis

HbA1c was measured by HPLC using ion-exchange chromatography (Bio-Rad Laboratories, USA, intra-and inter-assay CV 0.58% and 0.49%, respectively). All anterior pituitary hormones, plasma C-peptide and insulin were measured by the electrochemiluminescence immunoassay (ECLIA) (COBAS 600, Roche diagnostics, Germany). IGF-1 was measured by ECLIA (Dia-Sorin, Liaison, Germany). GIP (Total), GLP- 1 (Total) and glucagon were measured by sandwich ELISA method using kits manufactured by Millipore Corporation, Billerica, USA. The assay range for GIP, GLP-1 and glucagon assay were 4.2 to 2000 pg/ml, 4.1 to 1000 pM and 0.02 to 2 ng/ml respectively. The intra- and inter assay % CV for GIP, GLP-1 and glucagon assay was 6.7% & 6.1%, 2% & 12%, and 3.09% & 3.06%, respectively.

### Mixed meal test

The mixed meal test was done using a standardized mixed meal, 10 Kcal/kg of Ensure powder (Abbott Nutrition, Abbott Laboratories). The mixed meal used composed of 57% carbohydrate (sugar 14.7 gm/100 gm), 13.5% fat and 15.1% protein. After an overnight fast of 12 hours, fasting samples for glucose, insulin, C-peptide, GIP (total), GLP-1 (total) and glucagon were collected. The mixed meal (10 Kcal/kg) diluted in 300 ml of water was consumed by the patient in 10 minutes. Repeat samples were drawn at 30, 60, 90,120,150 and 180 minutes. Samples for blood glucose were collected in fluoride vials, while those for insulin and C-peptide were collected in EDTA vials. Blood samples for GIP, GLP-1 and glucagon were collected in pre-chilled EDTA-coated tubes containing aprotinin and an inhibitor of dipeptidyl peptidase IV. The samples collected for these hormones were immediately centrifuged at 4 °C and plasma was stored at −80 °C until analysis at a later date.

### Insulin secretion model

Basal β-cell function (BBCF) and postprandial β-cell function (PBCF) were analysed from C-peptide and glucose time-concentration profiles during the MMT using an insulin secretion model. M_0_ is an index of the BBCF and represents the ability of fasting glucose to stimulate β-cell. M_1_ is an index of PBCF and represents the ability of postprandial glucose to step up β-cell secretion. It equals the increment in secretion in response to a unit increment in glucose concentration^27^.

### Hyperinsulinemic euglycemic clamp

Insulin sensitivity was estimated by a hyperinsulinemic euglycemic clamp performed in the morning after an overnight fast of 12 hours. The HEC was done according to standard protocol. An intravenous cannula was inserted into the antecubital vein for infusion of insulin and 25% dextrose solutions. Another intravenous cannula was inserted in an anti-flow direction into the dorsal vein of the contralateral hand for collecting arterialized blood for measuring blood glucose. An insulin infusate of 300 mU/ml was prepared by adding regular human insulin (Eli Lilly & Co. Gurgaon, India) to 100 ml isotonic saline.

Insulin infusion was given at a constant rate of 40 mU/m^2^/min to raise the plasma insulin concentration to100 μU/mL. Blood samples for measuring glucose were collected at an interval of every 5 min and were analysed by the glucose oxidase method using a bedside glucose analyzer (GM9D, Analox instruments, London, UK). The glucose infusion rate was adjusted to maintain blood glucose at a steady state of 90 mg/dL. Insulin was measured in samples drawn at 10, 20, 40, 60, 80, 100 and 120 min. The amount of glucose metabolized by the individual, GDR, was calculated on the basis of the amount of glucose infused during the 40 to 120 min of the clamp, expressed as milligrams × (kilograms body weight × minute)^−1^. Insulin sensitivity was calculated as the amount of glucose metabolized per unit of plasma insulin (expressed as the mean insulin concentration during the 40 to 120 min of the clamp) × 100^28^.

### Indices of incretin-resistance

Insulin secretion in response to incretins is a surrogate evidence of preserved sensitivity of β-cells to circulating incretins. Hence, estimation of the ratio of insulin and C-peptide to GLP-1 and GIP may be an indication of incretin resistance. Therefore, we used the ratio of AUC for C-peptide to GLP-1 and GIP as a marker of incretin resistance.

### Histopathology

All 20 patients of acromegaly and an equal number of controls (n = 20) who were age, BMI and HbA1c matched were subjected to upper gastrointestinal endoscopy and sigmoidoscopy for taking duodenal and sigmoid biopsies, respectively. Immunohistochemistry (IHC) was performed on these tissue samples by peroxidase and anti-peroxidase technique. The primary antibodies used in the study were rabbit polyclonal anti-GIP (Abcam, ab48286) and anti-GLP-1 (Abcam, ab22625). The secondary antibodies used were anti-rabbit IgG (Vector Laboratories). The IHC procedure and the primary antibody titres were standardized using normal duodenal tissue of the patients who had undergone Whipple’s procedure. To quantify the positively stained cells, the images of the biopsies were analysed using Aperio image analysis software. Algorithm was tuned with the control tissue. Both the number of strong positively stained cells and the intensity of the strong positively stained cells were analyzed. Log2 fold change was calculated for patients of acromegaly in comparison to healthy controls.

### Statistical analysis

Insulinogenic index and Matsuda index were calculated using the standard formula (Δ Insulin_30 min–0 min_/Δ Glucose_30 min–0 min_) and [10,000/√ (FG_0_ × FPI_0_) (Mean Glucose x Mean Insulin)] respectively. The homeostasis model assessment (HOMA 2), a computer software was used to measure HOMA–IR, HOMA-β and HOMA-IS, using C-peptide. The area under the curve (AUC) was calculated using the trapezoid rule.

The data has been presented in mean and standard deviations along with mean ranks as data was small and normal distribution was not present. Further analysis of variables have been done using non-parametric tests of comparison: Kruskal Wallis with post hoc analysis was used for comparing all three groups (Healthy controls, Group A & B) and Mann Whitney U-test was used for comparing the two study groups (Group A & B). For the comparison of pre and post values of the same group non parametric Wilcoxon Signed Rank Test was used. Pearson’s correlation analysis was used to assess the correlations of different variables. A ‘p’ value less than 0.05 was considered significant.Analysis was conducted using IBM SPSS STATISTICS (version 22.0).

### Ethics approval and consent to participate

The study was performed according to the declaration of Helsinki and was approved by the Instiutional Ethics Committee of Postgraduate Institute of Medical Education and Research, Chandigarh, India. Written informed consent was obtained from all the patients to participate in the study.

## Results

### Study population characteristics

Twenty patients of active acromegaly (10 female, 10 male) and 10 age, BMI and HbA1c matched healthy controls were enrolled in the study. The mean age of the patients was 34.6 ± 9.0 yrs, mean GH and IGF-1 were 70.3 ± 66.6 ng/ml and 1086.3 ± 346.9 ng/ml, respectively. All patients harboured a pituitary macroadenoma and the median tumor volume before surgery was 3853.0 mm^3^ (1906.8–6407.9 mm^3^). Secondary hypothyroidism, hypocortisolism and hypogonadism were present in 8, 9 and 10 patients, respectively and they were replaced for respective hormone deficiencies accordingly.

Patients were divided into two groups based on their baseline glycaemic status. Group A included 10 patients (5 men) of acromegaly with euglycaemia, and group B had 10 patients (5 men) of acromegaly with hyperglycaemia. The HbA1c was significantly higher in group B as compared to the group A (9.6% ± 3.5 vs 5.4% ± 0.2, p = 0.001). However, the mean GH, IGF-1, tumor volume and anterior pituitary hormone status were comparable in both the groups (Table 1). Following surgery, only 8 patients achieved cure (6 in group A, 2 in group B), when evaluated 3 months after TSS. However, the mean GH, IGF-1 levels and tumor volume decreased significantly in both the groups following surgery.

**Table 1:** Baseline characteristics of healthy controls and patients of acromegaly.

Parameters	Group HC Healthy controls (n = 10) MEAN ± S.D. (Mean Rank)	Group A Acromegaly without Diabetes (n = 10) MEAN ± S.D. (Mean Rank)	Group B Acromegaly with Diabetes (n = 10) MEAN ± S.D. (Mean Rank)	Non parametric tests (Man Whitney/Kruskal Wallis) Chi square (p value) Post Hoc
Age (yrs)	29.7 ± 4.8 (12.10)	32.4 ± 10.5 (14.85)	36.8 ± 7.6 (19.55)	3.68 (p = 1.59)
Sex (n) M:F	5:5	5:5	5:5	0.0005 (p = 1.00) (Pearson Chi-square)
BMI (Kg/m^2^)	27.7 ± 2.4 (18.95)	26.5 ± 2.7 (13.10)	27.1 ± 3.9 (14.45)	2.69(0.260)
HbA1c (%)	5.0 ± 0.35 (07.15)	5.4 ± 0.24 (13.85)	9.6 ± 3.5 (25.50)	22.385 (0.0005***) HC < B***, A < B**
GH (basal) (ng/ml)	0.07 ± 0.07 (05.50)	74.8 ± 78.1 (20.30)	65.8 ± 56.9 (20.70)	19.52 (0.0005***) HC < A***, HC < B***
IGF 1 (ng/ml)	—	1198.9 ± 349.4 (12.50)	972.5 ± 319.8 (08.50)	30 (0.131)
Tumor volume (cu mm)	—	5132.4 ± 6288.7 (9.30)	7115.0 ± 8504.2 (11.70)	38 (0.364)

*P < 0.05, **P < 0.01, ***P < 0.001.

### Glucose, insulin and C-peptide

**Figure 1:**
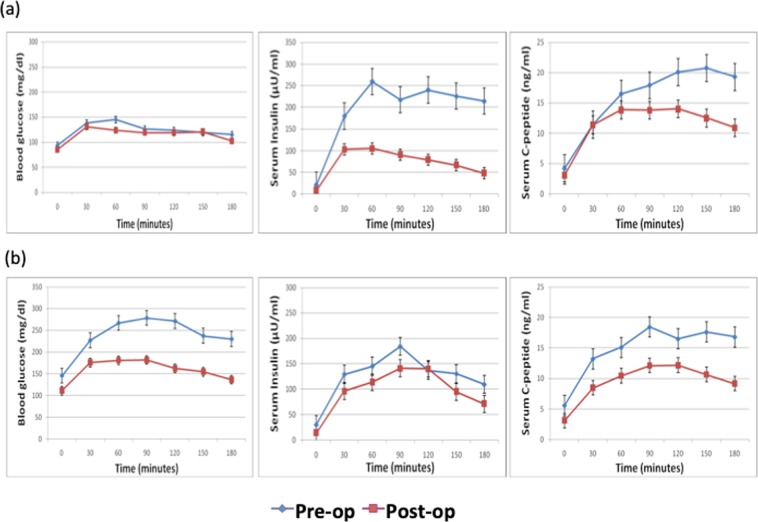
(**a**) Plasma glucose, insulin and C-peptide levels in Group A during the mixed meal test pre- and post-operatively. (**b**) Plasma glucose, insulin and C-peptide levels in Group B during the mixed meal test pre- and post-operatively.

At baseline the FPG and AUC for glucose were significantly higher in group B as compared to group A. While the FPI, C-peptide and their respective AUC were comparable between the study groups at baseline (Tables 2 and 3) (Fig. 1). After surgery, FPG, FPI and C-peptide decreased significantly in both the groups, though these could not attain statistical significance on intergroup comparison (Table 4). Further, the increase in M_1_ response could not attain significance in intra-and intergroup comparison (Table 5).

**Table 2:** Baseline biochemical characteristics of healthy controls and patients of acromegaly.

Parameters	Group HC Healthy controls (n = 10) MEAN ± S.D. (Mean Rank)	Group A Acromegaly without Diabetes (n = 10) MEAN ± S.D. (Mean Rank)	Group B Acromegaly with Diabetes (n = 10) MEAN ± S.D. (Mean Rank)	Non parametric tests (Kruskal Wallis) Chi square (p value) Post Hoc
FPG (mg/dl)	86.60 ± 06.55 (07.00)	93.63 ± 03.82 (14.20)	145.41 ± 49.19 (25.30)	21.933 (0.0005***) HC < B***, A < B**
FPI (µU/ml)	12.66 ± 04.07 (08.20)	20.80 ± 05.3 (19.80)	30.39 ± 23.24 (18.50)	10.423 (0.005**) HC < A**, HC < B*
FCP (ng/ml)	02.38 ± 0.57 (06.90)	04.23 ± 0.89 (20.15)	05.55 ± 03.87 (19.45)	14.359 (0.001***) HC < A***, HC < B**
HOMA-IR	1.71 ± 0.396 (05.60)	03.53. ± 01.01 (19.00)	04.12 ± 01.57 (21.90)	19.51 (0.0005***) HC < A**, HC < B***
HOMA-IS	62.08 ± 18.46 (25.40)	30.20 ± 07.86 (12.00)	26.72 ± 07.56 (09.10)	19.51 (0.0005***) HC** > A, HC > B***
Matsuda Index	3.8 ± 1.6 (24.30)	1.6 ± 0.5 (11.90)	1.6 ± 1.3 (10.30)	15.154 (0.001***) HC** > A, HC > B***
HOMA-β	153.86 ± 43.84 (14.60)	213.13 ± 48.76 (23.70)	11.97 ± 43.85 (08.20)	15.65 (0.0005***) A*** > B
Insulinogenic index	7.7 ± 5.7 (20.75)	4.4 ± 4.5 (15.80)	1.6 ± 1.0 (8.20)	10.733 (0.0005***) HC** > B
**Hyperinsulinemic Euglycemic Clamp**				
GDR (mg/kg/min)	—	2.57 ± 0.84 (13.70)	1.75 ± 0.62 (07.30)	18.00 (0.015**) (X)
IS (mg/kg/min/uU/ml)	—	04.48 ± 02.15 (11.00)	04.06 ± 02.66 (10.00)	45.00 (0.705) (X)

FPG - Fasting plasma glucose; FPI - Fasting plasma insulin; FPC - Fasting plasma c-peptide; HOMA-IR- Homeostatic model assessment of insulin resistance; HOMA-β -Homeostatic model assessment of β cell function; HOMA-IS- Homeostatic model assessment of insulin sensitivity; GDR: Glucose disposal rate; and IS- insulin sensitivity. *P < 0.05, **P < 0.01, ***P < 0.001.

**Table 3:** Comparison of the incretin and glucose – insulin responses during mixed meal test in healthy controls and patients of acromegaly.

Parameters	Group HC Healthy controls (n = 10) MEAN ± S.D. (Mean Rank)	Group A Acromegaly without Diabetes (n = 10) MEAN ± S.D. (Mean Rank)	Group B Acromegaly with Diabetes (n = 10) MEAN ± S.D. (Mean Rank)	Non parametric tests (Kruskal Wallis) Chi square (p value) Post Hoc
AUC-Glucose (mg/dL × min)	16136.0 ± 1550.5 (05.50)	22686.1 ± 2366.9 (15.70)	44039.7 ± 15218.7 (25.30)	25.30 (0.0005***) HC < A*, HC < B***, A < B*
AUC-Insulin (µU/ml × min)	17882.0 ± 6984.0 (09.80)	37175.3 ± 23608.2 (22.40)	23870.7 ± 14960.6 (14.30)	10.52 (0.005**) HC < A**
AUC C-peptide (ng/ml × min)	1355.5 ± 387.6 (06.80)	2962.8 ± 631.0 (22.40)	2758.5 ± 1704.7 (17.30)	16.33 (0.0005***) HC < A***, HC < B*
Fasting GIP (total) (pmol/L)	15.9 ± 5.2 (07.40)	32.1 ± 16.3 (17.30)	49.7 ± 30.0 (21.80)	14.00 (0.001***) HC < A*, HC < B***
AUC-GIP (pg/ml × min)	15241.0 ± 4139.6 (09.00)	19816.9 ± 4960.7 (16.00)	27383.7 ± 10058.2 (21.50)	10.13(2) (0.006**) HC < B**
Fasting GLP-1 (total) (pmol/L)	22.8 ± 9.7 (21.60)	9.3 ± 3.7 (09.30)	16.9 ± 10.9 (15.60)	09.76 (0.008**) HC > A**
AUC-GLP-1 (pmol/L × min)	8794.2 ± 2208.7 (23.70)	2782.9 ± 1388.0 (7.90)	5870.7 ± 4263.9 (14.90)	16.18 (0.0005***) HC > A***
Fasting Glucagon (pmol/L)	3.7 ± 1.4 (6.40)	10.4 ± 4.5 (15.30)	30.2 ± 13.4 (24.80)	21.85 (0.0005***) HC < B***, A < B*
AUC-Glucagon (pmol/L × min)	627.9 ± 71.5 (7.00)	3072.6 ± 2528.9 (16.10)	5296.6 ± 2170.1 (23.40)	17.42 (0.0005***) HC < B***
M_0_ × 10^−7^ (1/min)	−1.1 ± 7.9 (16.90)	−9.5 ± 86.8 (13.20)	−7.3 ± 35.4 (16.40)	1.040 (0.595)
M_1_ × 10^−7^ (1/min)	4.1 ± 7.1 (6.80)	81.2 ± 74.8 (21.50)	43.0 ± 32.9 (18.20)	15.352 (0.0005***) HC < A***, HC < B**

AUC - Area under curve; M_0_ – Basal β-cell function; M_1_ - postprandial β-cell function. *P < 0.05, **P < 0.01, ***P < 0.001.

**Table 4:** ∆ Change in glucose-insulin status, indices of IS and β-cell function after surgery.

Parameters	Group A (n = 10) Acromegaly without Diabetes			Group B (n = 10) Acromegaly with Diabetes			Wilcoxon Signed Rank Test for group A Z (P value)	Wilcoxon Signed Rank Test for group B Z (P value)	*Man Whitney U test* between Group A and Group B *Chi square* (*p value*)
	Pre-op Mean ± S. D. (Mean Rank)	Post-op Mean ± S. D. (Mean Rank)	Delta Change Mean ± S. D. (Mean Rank)	Pre-op Mean ± S. D. (Mean Rank)	Post-op Mean ± S. D. (Mean Rank)	Delta Change Mean ± S. D. (Mean Rank)			
FPG (mg/dl)	93.6 ± 3.8 (1.00)	85.2 ± 5.1 (6.00)	−8.4 ± 6.7 (11.90)	145.1 ± 49.2 (3.00)	111.0 ± 25.1 (6.13)	−36.7 ± 49.1 (09.10)	−2.701 (0.007**)	−2.191 (0.028*)	36.000 (0.290)
FPI (µU/ml)	20.8 ± 8.4 (0.000	8.6 ± 6.1 (5.50)	−15.9 ± 8.56 (8.40)	30.4 ± 23.4 (0.00)	14.4 ± 12.0 (5.50)	−9.6 ± 7.9 (12.60)	−2.803 (0.005**)	−2.803 (0.005**)	29.000 (0.112)
Fasting C-peptide (ng/ml)	4.2 ± 1.2 (1.00)	3.1 ± 1.0 (6.00)	−1.8 ± 1.6 (10.95)	5.5 ± 3.9 (3.00)	3.1 ± 1.7 (5.78)	−1.7 ± 1.3 (10.05)	−2.703 (0.007**)	−2.497 (0.013**)	45.500 (0.734)
HOMA-IR	3.5 ± 1.0 (0.00)	2.2 ± 0.7 (5.50)	−1.3 ± 1.1 (11.60)	4.1 ± 1.6 (6.00)	2.5 ± 1.5 (1.00)	−1.7 ± 1.1 (9.40)	−2.803 (0.005**)	−2.701 (0.007**)	39.000 (0.406)
HOMA-IS	30.2 ± 7.9 (5.50)	49.8 ± 14.1 (0.00)	19.6 ± 13.6 (11.60)	26.7 ± 7.6 (6.00)	60.3 ± 50.2 (1.00)	33.6 ± 48.5 (9.40)	−2.803 (0.005**)	−2.70 (0.007**)	45.000 (0.705)
Matsuda Index	1.6 ± 0.5 (5.50)	5.4 ± 3.3 (0.00)	3.9 ± 2.9 (10.00)	1.6 ± 1.4 (5.50)	4.1 ± 2.8 (0.00)	2.5 ± 2.5 (11.00)	−2.803 (0.005**)	−2.803 (0.005**)	33.000 (0.199)
HOMA-β	213.1 ± 48.8 (4.00)	186.2 ± 56.2 (6.50)	−26.9 ± 72.3 (12.200	112.0 ± 43.9 (6.50)	114.9 ± 32.0 (4.83)	2.9 ± 47.4 (8.80)	−1.172 (0.241)	−0.153 (0.878)	36.500 (0.307)
Insulinogenic index	4.4 ± 4.5 (7.00)	2.2 ± 1.6 (5.53)	−2.2 ± 3.9 (8.60)	1.6 ± 1.0 (5.33)	1.4 ± 0.9 (5.75)	−0.2 ± 0.8 (12.40)	−2.090 (0.037*)	−1.173 (0.241)	31.000 (0.151)
**Hyperinsulinemic Euglycemic Clamp**									
GDR (mg/kg/min)	2.7 ± 0.9 (5.89)	4.2 ± 1.2 (2.00)	1.6, 1.3 (11.00)	1.8 ± 0.6 (5.50)	3.1 ± 1.6 (0.00)	1.3 ± 1.3 (10.00)	−2.599 (0.009**)	−2.805 (0.005**)	45.000 (0.705)
IS (mg/kg/min/uU/ml)	4.5 ± 2.1 (5.50)	9.9 ± 3.9 (0.00)	5.4,3.2 (13.90)	4.1 ± 2.7 (6.000	6.3 ± 3.6 (1.00)	2.2 ± 2.2 (7.10)	−2.805 (0.005*)	−2.701 (0.007**)	16.000 (0.009**)

FPG - Fasting plasma glucose; FPI - Fasting plasma insulin; FPC - Fasting plasma c-peptide; HOMA-IR- Homeostatic model assessment of insulin resistance; HOMA-β -Homeostatic model assessment of β cell function; HOMA-IS- Homeostatic model assessment of insulin sensitivity; GDR: Glucose disposal rate; and IS- insulin sensitivity. *P < 0.05, **P < 0.01, ***P < 0.001.

**Table 5:** ∆ Change in glucose-insulin status and incretin parameters in response to MMT after surgery.

Parameters	Group A (n = 10) Acromegaly without Diabetes			Group B (n = 10) Acromegaly with Diabetes			Wilcoxon Signed Rank Test for group A Z (P value)	Wilcoxon Signed Rank Test for group B Z (P value)	*Man Whitney U test* between Group A and Group B *Chi square* (*p value*)
	Pre-op Mean ± S. D. (Mean Rank)	Post-op Mean ± S. D. (Mean Rank)	Delta Change Mean ± S. D. (Mean Rank)	Pre-op Mean ± S. D. (Mean Rank)	Post-op Mean ± S. D. (Mean Rank)	Delta Change Mean ± S. D. (Mean Rank)			
AUC-glucose (mg/dl × min)	22686.1 ± 2366.9 (3.25)	21198.4 ± 2161.8 (7.00)	−1487.7 ± 2766.9 (14.10)	44039.7 ± 15218.7 (2.00)	29343.6 ± 7263.4 (5.89)	−14696.1 ± 13672.3 (6.90)	−1.478 (0.139)	−2.599 (0.009**)	14.000 (0.007**)
AUC-Insulin (µU/ml × min)	37174.3 ± 23608.2 (0.00)	14169.7 ± 9093.2 (5.50)	−23005.6 ± 18053.7 (6.50)	23870.8 ± 14960.6 (4.50)	18858.9 ± 20142.9 (5.75)	−5011.8 ± 8189.4 (14.50)	−2.803 (0.005**)	−1.866 (0.059)	10.000 (0.002**)
AUC- C- peptide (ng/ml × min	2953.8 ± 626.4 (1.50)	2177.8 ± 608.4 (6.50)	−776.2 ± 620.6 (10.70)	2758.5 ± 1704.7 (0.00)	1800.9 ± 967.0 (5.50)	−957.6 ± 869.3 (10.30)	−2.497 (0.013*)	−2.803 (0.005**)	48.000 (0.880)
Fasting GIP (pmol/L)	32.1 ± 16.3 (2.50)	19.7 ± 10.0 (6.25)	−12.4 ± −16.8 (11.80)	49.7 ± 30.0 (7.50)	32.9 ± 19.1 (5.00)	−16.8 ± 31.8 (9.20)	−2.293 (0.022*)	−1.274 (0.203)	37.000 (0.326)
AUC-GIP (pmol/L × min)	19817.0 ± 4960.7 (1.50)	13300.5 ± 3142.3 (6.50)	−6486.5 ± 5636.1 (10.60)	27383.7 ± 10058.2 (8.00)	20943.8 ± 6757.0 (5.22)	−6439.2 ± 8844.5 (10.40)	−2.497 (0.033*)	−1.988 (0.047*)	49.000 (0.940)
Fasting GLP-1 (pmol/L)	9.3 ± 3.7 (6.60)	10.3 ± 5.7 (4.40)	1.0 ± 3.3 (11.40)	16.9 ± 10.9 (5.00)	14.3 ± 8.4 (6.00)	−2.6 ± 9.8 (9.60)	−0.561 (0.575)	−0.255 (0.799)	41.000 (0.496)
AUC-GLP1 (pmol/L × min)	2782.9 ± 1388.0 (6.14)	3594.0 ± 1718.8 (4.00)	811.1 ± 1458.7 (12.50)	5870.7 ± 4263.9 (5.00)	4579.7 ± 2718.7 (5.71)	−1291.1, ± 3301.5 (8.50)	−1.580 (0.114)	−1.274 (p = 0.203)	30.000 (0.131)
Fasting Glucagon (pmol/L)	10.4 ± 5.5 (3.50)	5.4 ± 2.7 (6.00)	−5.1 ± 5.8 (14.00)	30.2 ± 13.4 (1.00)	14.1 ± 4.5 (6.00)	−16.0 ± 11.1 (7.00)	−2.090 (0.037*)	−2.701 (0.007**)	15.000 (0.008*)
AUC Glucagon (pmol/L × min)	3072.6 ± 2528.9 (4.50)	1853.6 ± 1463.4 (5.75)	−1218.9 ± 1562.9 (12.30)	5296.6 ± 2170.1 (1.00)	2957.8 ± 881.7 (6.00)	−2338.8 ± 2108.5 (8.70)	−1.886 (0.059)	−2.701 (p = 0.007*)	32.000 (0.174)
M_0_ ×10^−7^ (1/min)	−9.5 ± 86.8 (6.67)	−30.9 ± 29.2 (5.50)	−21.4 ± 8.2 (9.90))	−7.3 ± 35.3 (6.67)	−15.2 ± 18.5 (5.00)	−7.9 ± 41.3 (11.10)	−0.764 (0.445)	−0.764 (0.445)	44.000 (0.650)
M_1_ ×10^−7^ (1/min)	81.2 ± 73.8 (4.67)	78.7 ± 29.9 (6.75)	−2.6 ± 68.5 (10.20)	43.0 ± 32.9 (6.20)	43.9 ± 20.6 (4.80)	0.9 ± 35.8 (10.80)	−0.051 (0.959)	−0.357 (0.721)	47.000 (0.821)

AUC - Area under curve; M_0_ - Basal β-cell function; M_1_ - postprandial β-cell function. *P < 0.05, **P < 0.01, ***P < 0.001.

### Indices of Insulin sensitivity (IS) and β-cell function

The indices of IS were reduced to comparable levels in patients of acromegaly both with and without hyperglycaemia, whereas the indices of β-cell function (HOMA-β) were significantly lower in those with hyperglycaemia as compared to those with euglycaemia (Table 2). After surgery the indices of IS improved significantly in both the groups, while the indices of β-cell function improved significantly (represented as decline in β-cell indices) only in those with euglycaemia (Table 4). Further, the degree of insulin resistance (HOMA-IR) correlated with GH (r = 0.76, p = 0.03) and IGF-1 (r = 0.72, p = 0.03).

### Incretin hormones

#### Fasting GIP (total) levels and GIP response during MMT

**Figure 2:**
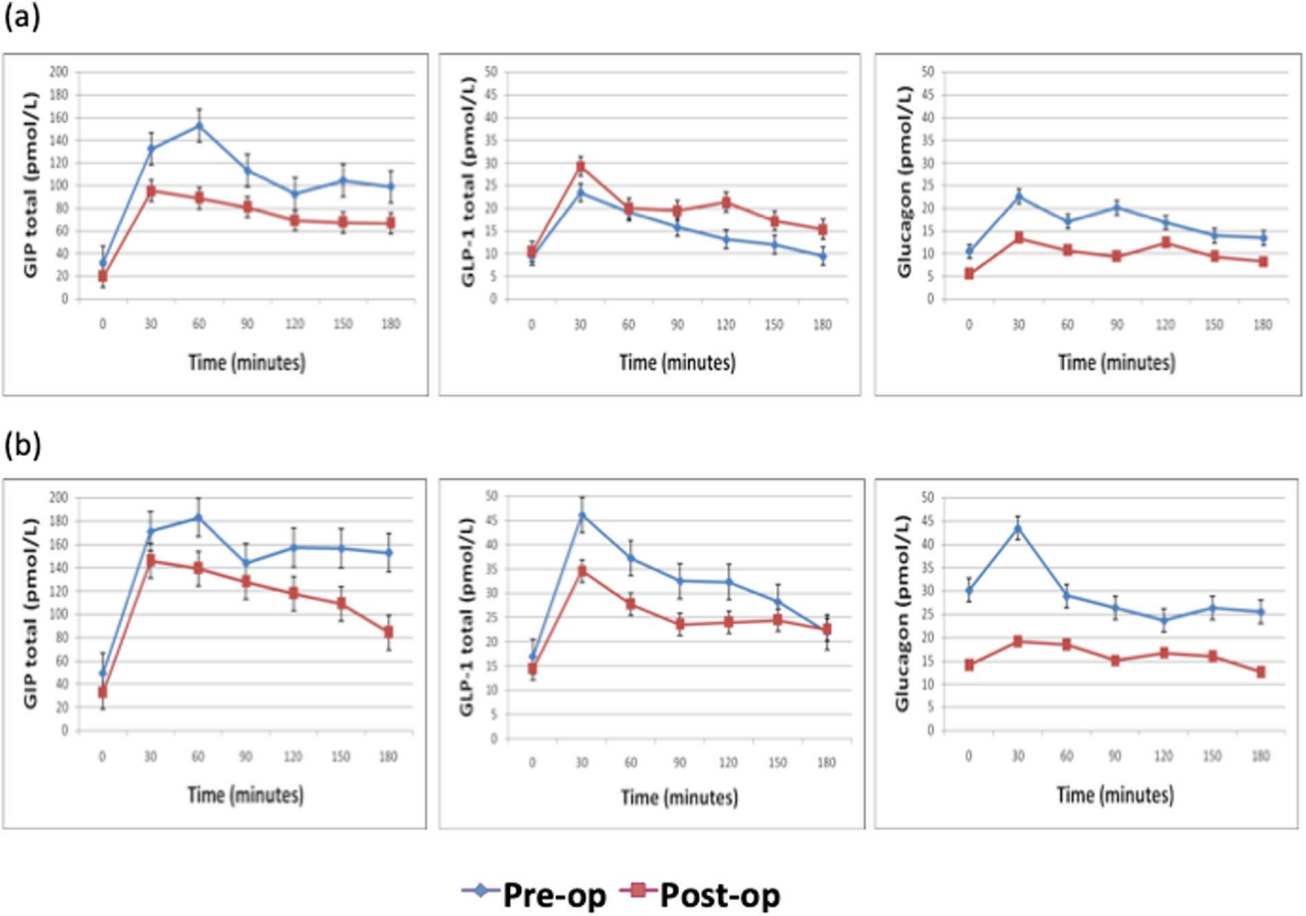
(**a**) GIP, GLP-1 and glucagon response in Group A during the mixed meal test pre- and post-operatively. (**b**) GIP, GLP-1 and glucagon response in Group B during the mixed meal test pre- and post-operatively.

The fasting GIP levels and its AUC before surgery (Table 3) were significantly higher in patients of acromegaly as compared to healthy subjects. The AUC for GIP was modestly higher in patients of acromegaly with diabetes than in those without diabetes (Table 3). Post-operatively, the fasting GIP levels in group A and AUC for GIP in both groups decreased significantly (Table 5) (Fig. 2), and correlated with reduction in HOMA-IR (r = 0.64,p = 0.047 and r = 0.78, p < 0.001), GH (r = 0.63, p = 0.04 and r = 0.69, p = 0.03) and IGF-1 levels (r = 0.75, p = 0.04 and r = 0.84, p < 0.001).

#### GLP-1(total) levels and GLP-1 response during MMT

The fasting GLP-1 levels and its AUC were lower in patients of acromegaly in comparison to healthy subjects. However, the AUC for GLP-1(total) during the MMT before surgery, was modestly higher in those with diabetes as compared to those without diabetes though still lesser than the healthy controls (Table 3). Following surgery, there were non-significant alterations in fasting GLP-1 and its AUC levels in both group A and B (Table 5) and (Fig. 2).

#### Glucagon levels and Glucagon response during MMT

The fasting glucagon levels and its AUC at baseline were significantly higher in patients of acromegaly than the levels seen in healthy controls (Table 3). The levels before surgery were significantly higher in group B in comparison to the patients in group A (Table 3). Further, the fasting glucagon levels in both the groups and its AUC in group B decreased significantly (Table 5) (Fig. 2) following surgery.

#### Correlation between GIP and glucagon levels

Analysis of the correlation between GIP and glucagon levels in individual patients was not found to be significant. However, when the data of all the patients of acromegaly were pooled together, there was a rank order correlation between GIP and glucagon levels (r 0.73, p < 0.001). Using Youden’s J test, a GIP level of 314 pmol/L could predict hyperglycemia (≥140 mg/dl) with sensitivity of 65% and specificity of 100%. When the cut-off for glucose was raised to ≥200 mg/dl, it corresponded with GIP levels of 432 pmol/L with 100% sensitivity and 77% specificity.

#### Indices of Incretin resistance

Ratio of AUC for C-peptide/GIP was significantly higher in patients with acromegaly as compared to healthy controls. However, patients of acromegaly with diabetes had a significantly lower C-peptide/GIP ratio as compared to those without diabetes despite having markedly increased GIP levels and moderately preserved β-cell function suggesting GIP resistance (Table 6). Further ratio of AUC for C-peptide/GLP-1 was higher in patients with acromegaly as compared to controls predominantly because of decrease in GLP-1 levels (Table 6).

**Table 6:** Indices of incretin response in healthy controls and patients of acromegaly with and without diabetes.

Parameters	Group HC Healthy controls (n = 10) MEAN ± S.D. (Mean Rank)	Group A Acromegaly without Diabetes (n = 10) MEAN ± S.D. (Mean Rank)	Group B Acromegaly with Diabetes (n = 10) MEAN ± S.D. (Mean Rank)	Non parametric tests (Man Whitney/Kruskal Wallis) Chi square (p value) Post Hoc
AUC (C-peptide/GLP1) (pmol/L × min)	55.7 ± 25.7 (6.00)	467.2 ± 321.5 (24.10)	190.4 ± 96.5 (16.40)	21.293 (0.0005***) HC < A***, HC < B*
AUC (C-peptide/GIP) (pmol/L × min)	30.3 ± 8.7 (10.90)	50.4 ± 10.3 (22.90)	43.7 ± 45.9 (12.70)	10.808 (0.004*) HC < A**, B < A*

*P < 0.05, **P < 0.01, ***P < 0.001.

### Histopathology

**Figure 3:**
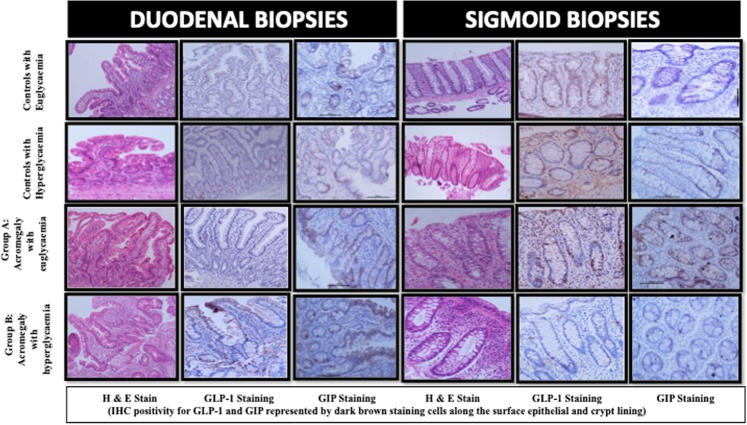
Photomicrographs of H & E stain and IHC staining (GLP-1 and GIP) of the duodenal and sigmoidal biopsies of the study and control groups showing a lower number and intensity of GIP staining cells in patients of acromegaly with diabetes. (IHC positivity for GIP and GLP-1 represented by the dark brown staining cells along the surface epithelial and crypt lining).

The H & E stained sections of all biopsies in the different groups showed similar histology. The IHC staining done for GIP and GLP-1 antibodies in the normal control was noted for the baseline interpretation. Distribution of the positively stained cells was taken into account while interpreting the different study groups. The number and intensity of GIP secreting cells were higher in healthy controls as compared to patients of acromegaly with diabetes (p = 0.009 and 0.003, respectively) (Fig. 3 and Supplementary Fig. 1). However, the staining pattern of the GLP-1 positive cells in the duodenal and colonic biopsies of the study group was similar to the control groups. The photomicrographs of the immunostaining with GIP and GLP-1 are shown in Fig. 3.

## Discussion

The present study demonstrated that the various indices of insulin sensitivity are reduced to comparable levels in patients of acromegaly irrespective of the presence or absence of hyperglycaemia. The indices of β-cell function however are significantly lower only in those with hyperglycaemia despite higher GIP levels. Following surgery, the indices of IS improved significantly both in patients of acromegaly with and without diabetes, whereas the indices of β-cell function improved significantly only in those with euglycaemia but not in patients with hyperglycaemia suggesting ‘β-cell exhaustion’ in patients of acromegaly with diabetes. IHC positivity for GIP was lower in the duodenal and colonic biopsies in patients of acromegaly, possible because of higher K-cell turnover. However IHC positivity for the GLP-1 staining cells was comparable in both the study and control groups irrespective of the glycaemic status.

Disruption in the glucose-insulin homeostasis is frequently present in patients of acromegaly. GH is a potent antagonist of the insulin action on carbohydrate metabolism and induces insulin resistance (IR) which is considered to be the major pathogenetic mechanism responsible for glucose intolerance seen in patients with acromegaly^4^. Studies done in the past have demonstrated unequivocal evidence of reduced insulin senstivity in patients of acromegaly. Foss *et al*. demonstrated that the glucose uptake in the forearm muscle and non-oxidative metabolism of glucose after the ingestion of 75-g glucose were significantly impaired preoperatively in patients of acromegaly^29^. Moller *et al*. and Wasada *et al*. using the hyperinsulinaemic euglycaemic clamp demonstrated a decreased rate of glucose infusion in patients with active acromegaly^30,31^. A study by Kim *et al*. had demonstrated that the indices of IS improved after tumor resection in patients of acromegaly^32^. In the present study the various indices of IS (HOMA-IR, HOMA-IS, Matsuda index and glucose disposal rate) were suppressed at baseline and improved significantly following surgery. Further, these indices of IS were reduced to comparable levels both in patients with euglycaemia and hyperglycaemia, despite of different glycaemic status of the two groups, indicating that the IR alone does not determine the glucose tolerance status in patients with acromegaly. Before surgery, the degree of insulin resistance (IR) correlated with the GH and IGF-1 levels, and postoperatively the significant decrease in GH and IGF-1 levels were accompanied with significant improvement in indices of IS indicating that elevated GH/IGF-1 are the main determinants of IR seen in patients with active acromegaly.

Before surgery, the β-cell indices (HOMA-β and insulinogenic index) were significantly higher in patients of acromegaly with euglycaemia than in those with hyperglycaemia. Following surgery, β-cell indices improved significantly in those with euglycaemia, while there was a non-significant alteration in those with hyperglycaemia. The patients of acromegaly with normal glucose tolerance could maintain euglycaemia even in the face of significantly increased insulin resistance due to the stimulatory/cytotrophic effect of elevated GIP and GH/IGF-1 on β-cells and/or as a part of innate β-cell reserve. In contrast, the indices of β-cell function were significantly lower in patients of acromegaly with hyperglycaemia, despite significantly higher GIP levels, with comparable IR indices and GH/IGF-1 levels. The prolonged GH-induced β-cell stimulation in these patients ultimately led to “β-cell exhaustion” resulting in hyperglycaemia. Our results are consistent with previous study by Kasayama *et al*. who showed that IS is reduced to similar extent in acromegalic patients irrespective of their glycaemic status^22^. The compensatory hyperfunction of β-cells counterbalances the reduced IS in those with euglycaemia, but not in those with hyperglycaemia. These results help us to infer that the residual pancreatic β-cell function determines the glucose tolerance status in patient with acromegaly rather than IR alone.

The fasting GIP levels and the AUC for GIP were higher at baseline in patients with acromegaly as compared to healthy subjects both in the study and in meta-analysis of various studies done in past^33^, and decreased significantly following surgery. The fasting GIP levels and its AUC were higher in those with hyperglycaemia than in those with euglycaemia. Following surgery, the AUC for GIP decreased significantly in both the groups. Our findings are consistent with a previous study by Peracchi *et al*., who had shown that GIP levels are increased both in fasting and post-prandial states in patients with acromegaly^23^. However, the study population included only patients of acromegaly with euglycaemia and did not study the effect of surgery on it. The present study is the first to document GIP levels in patients of acromegaly with different glycaemic status, both before and after surgery. Higher GIP levels in patients of acromegaly could have possibly resulted from the direct cytotrophic effect of elevated GH/IGF-1 on K-cells. Regazzo *et al*. have also proposed an existence of GH/IGF-1-GIP axis and our findings also concur the existence of such an axis leading to an increased GIP levels in patients of acromegaly^34^. A still higher levels of GIP in patients of acromegaly with hyperglycaemia as compared to those with euglycemia could possibly have resulted from hyperglycaemia-induced desensitization of GIP receptors on β-cells (GIP resistance), as also seen in patients with T2DM^35^.This was further substantiated by significantly lower AUC C-peptide/GIP ratio in patients of acromegaly with diabetes as compared to those with euglycaemia.

The GLP-1 levels in patients with acromegaly before surgery were lower in comparison to healthy subjects in our study but similar to the levels seen in meta-analysis of GLP-1 levels in patients with T2DM^36^. However, the AUC for GLP-1 was higher in patients of acromegaly with hyperglycaemia than in those with euglycaemia. Following surgery there was no significant alteration in either the fasting GLP-1 levels or it’s AUC in both the groups. The GLP-1 levels in patients of acromegaly have not been measured previously. Ours is the first study to document GLP-1 levels in patients of acromegaly, both before and after surgery. Failure of the GLP-1 levels to rise in patients of acromegaly, akin to GIP levels, can possibly be explained by the fact that GLP-1 is known to inhibit glucagon secretion and vice versa high levels of glucagon, seen in patients of acromegaly in our study, could have inhibited GLP-1 secretion^37^_._ Further, the insulin resistance has also been implicated to impair GLP-1 secretion^38^, and the inherent state of elevated insulin resistance associated with acromegaly could have impaired GLP-1 rise in these patients.

The fasting glucagon levels and its AUC observed in patients of acromegaly in our study were significantly higher than those observed in healthy controls in our study and in meta-analysis of various studies done in healthy controls^39^. Our findings are in conformity with Yutaka *et al*. who had documented higher glucagon levels in patients of acromegaly^24^. The glucagon levels before surgery were significantly higher in patients with hyperglycaemia than in euglycaemic subjects with acromegaly, and the levels decreased significantly after surgery in both the groups in parallel to the significant reduction in IR and GH/IGF-1 levels. The higher glucagon levels in patients of acromegaly could have possibly resulted from the direct cytotrophic effect of elevated GH/IGF-1 on pancreatic α-cells. Further, the GIP receptors are present on the pancreatic α-cells and GIP is known to stimulate glucagon secretion from α-cells^40^. The elevated GIP levels observed in patients of acromegaly in our study could have also contributed to hyperglucagonemia seen in these patients. Moreover, these patients had significantly higher fasting glucagon levels even in the face of elevated fasting plasma insulin, suggesting impaired cross-talk between α and β-cells due to insulin resistance. Ferrannini *et al*. had earlier shown using HEC that IR is independently associated with elevated fasting glucagon concentrations, possibly as a result of α-cell insulin resistance^41^. Hyperglucagonemia in patients of acromegaly and the lack of appropriate suppression of glucagon in post-prandial states is an outcome of exaggerated α-cell response to glucose and impaired insulin-mediated inhibition of α-cell through its paracrine action.

The pattern of expression of GIP and GLP-1 in the duodenal and colonic biopsies of patients with acromegaly has not been studied previously. We observed that both the number and intensity of GIP staining cells in the duodenal and colonic biopsies of patients of acromegaly with diabetes was significantly lower than the healthy controls. The GIP staining in these patients was lower despite increased circulating GIP levels indicating increased K-cell turn-over in patients of acromegaly with diabetes. The GLP-1 staining however was comparable in both patients of acromegaly and healthy controls.

The strengths of our study are a comprehensive assessment of the incretin-axis in patients with active acromegaly, inclusion of healthy controls, and corelation of the serum levels of incretin hormones with the expression of K and L cells in the intestine of these patients. A few limitations of our study includes a small sample size and non-availability of objective methods to assess the functionality of intestinal neuroendocrine cells.

## Conclusion

Impaired insulin sensitivity is innate to state of GH excess. The magnitude of insulin resistance induced by chronic GH exposure is independent of the glycaemic status and emergence of hyperglycaemia is an outcome of “β-cell dysfunction”, GIP resistance and hyperglucagonemia. These observations tender novel insights into the pathogenesis of dysglycaemia in acromegaly and pave the way for incretin-based therapies for the management of diabetes in these patients.

## Supplementary information

Supplementary Information

## Data Availability

The datasets supporting the conclusions of this work are included in the article.
