# Supplementary material for: Glucose-dependent Insulinotropic Polypeptide (GIP) Resistance and β-cell Dysfunction Contribute to Hyperglycaemia in Acromegaly
Source: Sci Rep. 2019 Apr 4;9:5646. doi: 10.1038/s41598-019-41887-7 (PMC6449401; doi:10.1038/s41598-019-41887-7)
Supplement: Supplementary file 1 — Supplementary Information [file 41598_2019_41887_MOESM1_ESM.pdf]

Glucose-dependent Insulinotropic Polypeptide (GIP) Resistance and β-cell Dysfunction Contribute to Hyperglycaemia in Acromegaly

Vikram Singh Shekhawat[1], Shobhit Bhansali[1], Pinaki Dutta[1], Kanchan Kumar Mukherjee[2], Kim Vaiphei[3], Rakesh Kochhar[4], Saroj K Sinha[4], Naresh Sachdeva[1], Anura V Kurpad[5], Kishor Bhat[5], Sunder Mudaliar[6] , Anil Bhansali[1*]

[1]Department of Endocrinology, [2]Department of Neurosurgery, [3]Department of Pathology,[4]Department of Gastroenterology, PGIMER Chandigarh, Sector 12, Chandigarh, 160012, India, [5]Department of Physiology, St. John's Medical College, Bangalore, India, [6]Department of Medicine, University of California, San Diego, La Jolla, California, USA

Correspondence:

Dr Anil Bhansali

Professor and Head, Department of Endocrinology,PGIMER, Chandigarh, India.

Pincode 160012 ,Telephone +91 0172 2756583.

Email : anilbhansaliendocrine@gmail.com.

Supplementary Table 1: Δ Change in glycaemic status after surgery

Parameters	Acromegaly Cured (n = 8) Mean± S.D. (Mean Rank)	Acromegaly Not Cured (n = 12) Mean± S.D. (Mean Rank)	*Man Whitney U test* *Chi square* (P value)
FPG (mg/dl)	-35.4± 52.4 (9)	-13.9± 20.6 (11.50)	0.857(0.355)
FPI (μU/ml)	-14.8±8.9 (8.75)	-11.4±8.7 MR=11.67	1.167(0.280)
Fasting C-peptide (ng/ml)	-2.0±1.6 (9.88)	-1.6±1.2 (10.92)	0.149 (0.700)
HOMA-IR	-1.8± 1.2 (9.13)	-1.2±1.0 (11.42)	0.720 (0.396)
HOMA- IS	27.5±15.4 (12.75)	25.9±44.8 MR=9	1.929(0.165)
Matsuda Index	4.9±2.9 (14.25)	2.0±1.9 (8)	1.168 (0.021*)
HOMA-β	-9.4±89.5 (11.50)	-13.8±37.7 (9.83)	0.381 (0.537)
Insulinogenic index	-2.2±3.9 (8.75)	-0.5±1.9 (11.67)	1.168 (0.280)
Hyperinsulinemic Euglycemic Clamp(HEC)			
GDR (mg/kg/min)	1.7±1.5 (11.38)	1.3±1.2 (9.92)	0.292 (0.589)

**IS (mg/kg/min/uU/ml)**	**5.3,3.2 (13.31)**	**2.9,2.8 (8.63)**	**3.016 (0.082)**

* Significant difference between the groups

FPG- Fasting plasma glucose; FPI- Fasting plasma insulin; FPC- Fasting plasma c-peptide; HOMA-IR- Homeostatic model assessment of insulin resistance; HOMA-β -Homeostatic model assessment of β cell function; HOMA-IS- Homeostatic model assessment of insulin sensitivity; GDR: Glucose disposal rate; and IS- insulin sensitivity.

Supplementary Table 2 : Δ Change in glycaemic status and incretin parameters after surgery during MMT

Parameters	Acromegaly Cured (n = 8) Mean± S.D. (Mean Rank)	Acromegaly Not Cured (n = 12) Mean± S.D. (Mean Rank)	*Man Whitney U test* *Chi square* (P value)
AUC-glucose (mg/dl × min)	-8586.0±15515.5 (11.13)	-7762.5±9220.7 (10.08)	0.149 (0.700)
AUC-Insulin (μU/ml×min)	-22551.9±21332.1 (7.50)	-8313.3±9521.9 (12.50)	3.429 (0.064)
AUC- C- peptide (ng/ml x min)	-1019.9±486.4 (8.25)	-765.5±877.3 (12.00)	1.929 (0.165)
Fasting GIP(pmol/L)	-18.0±24.1 (10.25)	-12.3±26.1 (10.67)	0.024 (0.877)
AUC-GIP (pmol/L × min)	-9654.1±5919.1 (7.75)	-4335.9±7440.2 (12.33)	2.881 (0.090)
Fasting GLP-1 (pmol/L)	0.2±3.7 (9.88)	-1.4±9.1 (10.92)	0.149 (0.700)
AUC-GLP1 (pmol/L × min)	731.3±1826.2 (11.38)	-887.5±3066.3 (9.92)	0.292 (0.589)
Fasting Glucagon (pmol/L)	-8.1± 5.8 (11.75)	-12.2±12.4 (9.67)	0.595 (0.440)
AUC Glucagon (pmol/L× min)	-1295.3±1554.1 (11.63)	-2101.2±2094.3 (9.75)	0.482 (0.487)
M_0 x10^{-7} (1/min)	-33.8±87.8	-1.8±39.6	1.006

	**MR=12.63**	**MR=9.08**	**(0.316)**
**M_1 x10^{-7} (1/min)**	**0.9±9.1**<br>**(8.88)**	**-0.9±5.4**<br>**(11.58)**	**1.720**<br>**(0.190)**

* Significant difference between the groups

AUC- Area under curve; M_0 – Basal β-cell function; M_1- postprandial β-cell function.

Figure Legends:

Supplementary Figure 1: The bar diagram showing a significantly lower number and intensity of GIP staining cells in patients of acromegaly with diabetes as compared to healthy controls.

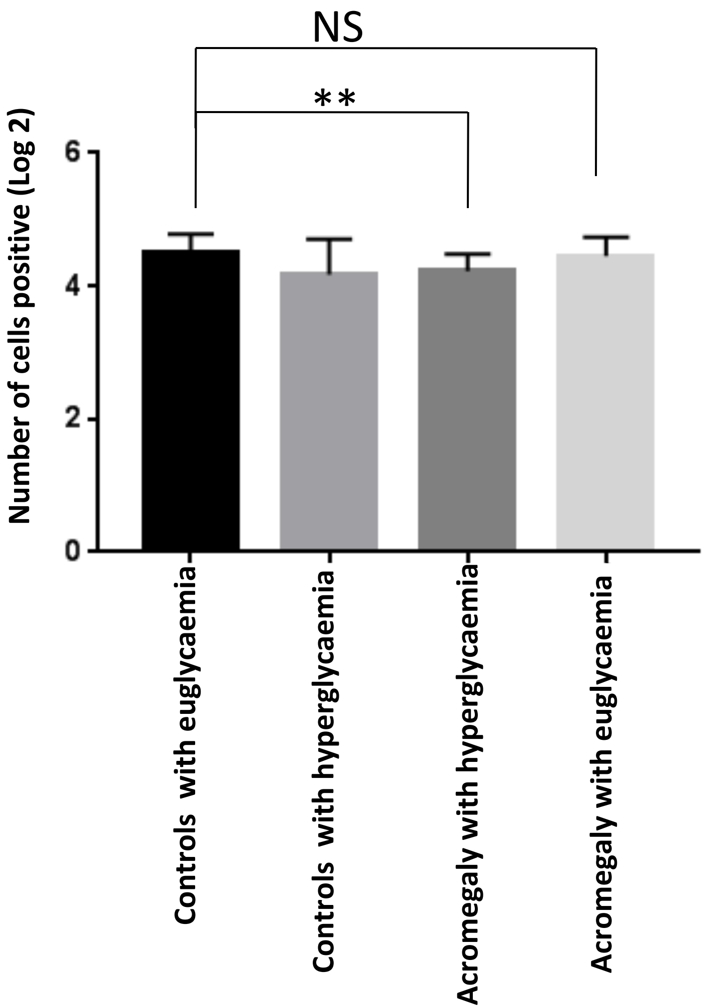

Number of cell positive (Log2)		P-value
Controls with euglycaemia	Acromegaly with hyperglycaemia	0.009**
	Acromegaly with euglycaemia	0.4
Controls with hyperglcaemia	Acromegaly with hyperglycaemia	0.54
	Acromegaly with euglycaemia	0.75
Acromegaly with hyperglycaemia	Acromegaly with euglycaemia	0.09

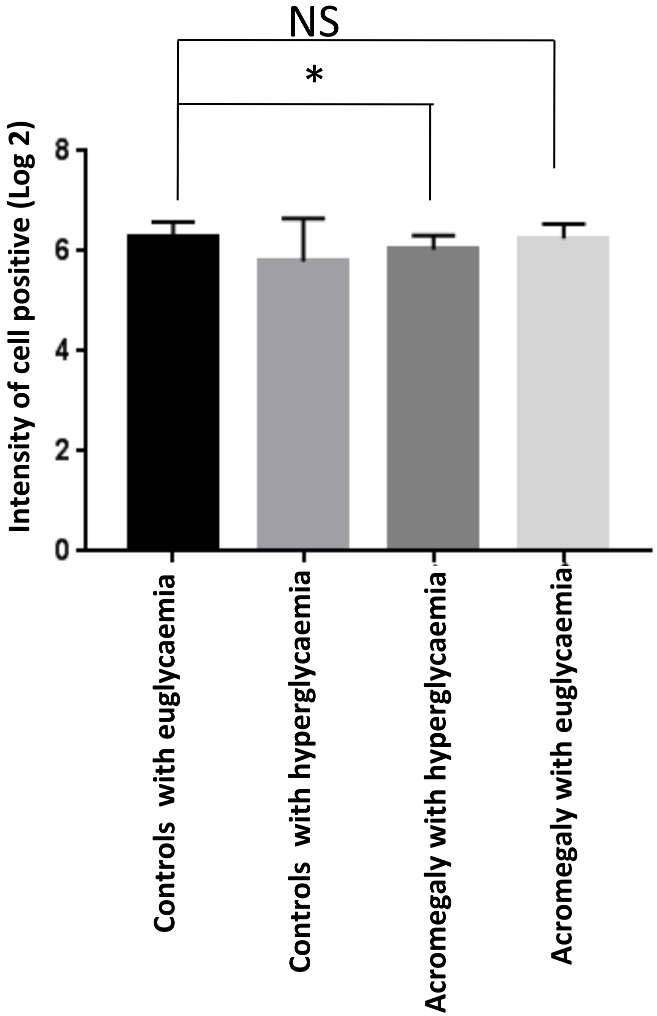

Intensity of cell positive (Log 2)		P-value
Controls with euglycaemia	Acromegaly with hyperglycaemia	0.03*
	Acromegaly with euglycaemia	0.455
Controls with hyperglcaemia	Acromegaly with hyperglycaemia	0.6526
	Acromegaly with euglycaemia	0.5848
Acromegaly with hyperglycaemia	Acromegaly with euglycaemia	0.1406
